# Associations of Social Support with Sexual Practices, Health Behaviours, and Health Outcomes Among Adolescent Girls and Young Women: Evidence From a Longitudinal Study in KwaZulu-Natal, South Africa

**DOI:** 10.1007/s12529-023-10199-6

**Published:** 2023-07-21

**Authors:** Dick Durevall, Richard G. Cowden, Sean Beckett, Ayesha B. M. Kharsany, Lara Lewis, Gavin George, Cherie Cawood, David Khanyile, Kaymarlin Govender

**Affiliations:** 1 Department of Economics, School of Economics, University of Gothenburg, Gothenburg, Sweden; 2 Human Flourishing Program, Harvard University, Cambridge, MA USA; 3 Health Economics and HIV and AIDS Research Division (HEARD), University of KwaZulu-Natal, Durban, South Africa; 4 Centre for the AIDS Programme of Research in South Africa (CAPRISA), University of KwaZulu-Natal, Durban, South Africa; 5 School of Laboratory Medicine and Medical Science, Nelson R Mandela School of Medicine, University of KwaZulu-Natal, Durban, South Africa; 6 Epicentre AIDS Risk Management (Pty) Limited, Cape Town, South Africa

**Keywords:** Adolescents, Female, HIV risk behaviours, HIV, Health risk behaviours, Social support, Outcome-wide approach

## Abstract

**Background:**

Several studies have reported on the benefits of social support for health behaviour, including risky sex. Social support may thus be an important resource for promoting individual health and well-being, particularly in regions where HIV rates are high and healthcare resources are scarce. However, prior research on the implications of social support for the health behaviour of young women has yielded mixed and inconclusive findings. Using prospective data from young women in South Africa, this study examines the associations of social support with subsequent sexual practices, health behaviour, and health outcomes.

**Method:**

We used two rounds of longitudinal data from a sample of *n* = 1446 HIV-negative emerging adult women, aged 18 to 29 years, who participated in a population-based HIV study in KwaZulu-Natal, South Africa. Applying the analytic template for outcome-wide longitudinal designs, we estimated the associations between combinations of social support (i.e. tangible, educational, emotional) and ten HIV risk–related outcomes.

**Results:**

Combinations of tangible, educational, and emotional support, as well as tangible support by itself, were associated with lower risk for several outcomes, whereas educational and emotional support, by themselves or together, showed little evidence of association with the outcomes.

**Conclusion:**

This study highlights the protective role of tangible support in an environment of widespread poverty, and the additional effect of combining tangible support with non-tangible support. The findings strengthen recent evidence on the benefits of combining support in the form of cash and food with psychosocial care in mitigating risk behaviours associated with HIV and negative health outcomes among young women.

**Supplementary Information:**

The online version contains supplementary material available at 10.1007/s12529-023-10199-6.

## Introduction

Social support has emerged as a factor that can have substantial health benefits [1–6]. In contexts where social-structural disadvantages (e.g. poverty, education, inequalities) pose significant challenges to many people, psychosocial and economic supports may be important resources for promoting individual health and well-being among vulnerable populations such as young women [7–10]. Using prospective data from a sample of young women living in a high-prevalence HIV region in South Africa, this study examines associations between different combinations of social support and a range of sexual behaviour, health behaviour, and health outcomes.

There are three primary theoretical perspectives regarding how social support influences health outcomes: the stress and coping perspective, the social constructionist perspective, and the relationship perspective [10]. These perspectives differ in their explanations of how social support impacts health outcomes. According to the stress and coping perspective, social support helps individuals cope with stressful situations by enhancing their capacity to manage stress and reducing the negative impact of stress on their health. The social constructionist perspective, on the other hand, posits that social support improves an individual’s self-esteem and self-regulation, ultimately contributing to improved health outcomes. Finally, the relationship perspective suggests that social support promotes stronger social ties, which can either positively or negatively affect health outcomes depending on the norms and attitudes of the social group [10]. Thus, the relationship between social support and health is contingent on both the form of support and the type of health outcome.

Several studies have empirically documented associations between social support and behaviours related to risky sex and HIV infection. One review of empirical studies focusing on the relationship between various forms of social support and sex-related risk behaviours found that higher levels of social support are related to fewer sex-related risk behaviours among female sex workers, people living with HIV, and heterosexual adults, while noting inconsistent results for adolescents [10]. However, the evidence in Africa is limited and mixed. Out of the 40 studies reviewed, only two reported separate regression results for African women. One of those studies found that social support was correlated with consistent condom use among HIV-positive South African women [11]. In the other study, social support among Zimbabwean women was associated with fewer sexual partners, but there was no evidence of an association with condom use [12]. Two other studies included African men and women; one found that social support was unrelated to consistent condom use among HIV-positive Ugandans [13], and the other did not find evidence of an association between social support and high-risk sex (more than one partner in the past year or no condom use) among adolescents in rural Kenya [14]. In a more recent study, social support among 15–19-year-old adolescent girls living in an impoverished area of South Africa was not associated with ever having had sex or condom use, but it was associated with fewer sex partners [15].

Some studies focus on social protection, in the form of cash transfers, and sexual risk behaviours, with the findings painting a picture that is comparable to the literature on social support [16]. Although social protection is provided by governments and organizations and does not create the same social relationships as social support, it is similar to social support in the sense that it can alleviate poverty. Moreover, it sometimes includes non-tangible components, such as improved caregiver care. One study found that South African adolescent girls (aged 10–18 years) living in households with state-provided child-focused cash transfers were less likely to have had transactional and age-disparate sex than their peers, but were equally likely to have had unprotected sex, multiple partners, and sex after drinking alcohol or taking drugs [17]. The findings of studies that analyse combinations of cash transfers and other types of support, such as caregiver care and psychological support, are more promising. Cluver et al. [18] analysed the impact of integrated cash and social support from caregivers and schools to South African girls (aged 10–18 years) on HIV risk, measured by an index of a number of indicators. Both cash and social support had independent effects on HIV risk, but the combined effect was substantially stronger. Using the same data, Cluver et al. [19] showed that social support had the strongest benefits for the most vulnerable girls, as measured by structural and psychosocial factors. In a study by Stoner et al. [16], receiving a child support grant (i.e. a government cash transfer) and caregiver care were not independently associated with HIV incidence among 13–20-year-old South African females, but they were associated with reduced HIV incidence when combined.

Few attempts have been made to evaluate why social support affects risky sex and HIV infection. However, a study on South African girls found that social protection, in the form of cash transfers, and care (primarily positive parenting and good parental monitoring) were each indirectly associated with lower HIV risk behaviour by reducing psychosocial problems, whereas cash was directly associated with reduced HIV risk behaviour [19]. A qualitative study in Tanzania found that cash transfers combined with a financial education programme was successful at building agency and improving self-esteem among girls and young women aged 15–23 years, which may have allowed them to refuse unwanted sex partners [20]. Similarly, a qualitative study of Tanzanian girls and young women indicated that cash may lead to empowerment, conceptualized as “independence” and “hope and aspiration”, which appeared to give them more authority to negotiate safe sexual behaviours. This may reduce the number of sexual partners and economic reliance on transactional sex [21].

There are several potentially relevant reasons for the diverse range of findings in the prior empirical literature in this area, such as differences in conceptualization and measurement of social support, the choice of outcomes, and the characteristics of participants and the contexts in which they live [22]. However, a key limitation of most studies is the reliance on cross-sectional data, which typically are susceptible to biases. Out of the 40 studies reviewed by Qiao et al. [10], only four used longitudinal data and they were all from the USA, which differs from the African context where social-structural disadvantages are more pervasive. In addition, most prior studies have focused on a single or narrow set of outcomes, which provides an incomplete picture of how social support might be related to different outcomes of interest. By including a broader range of outcomes, one may obtain a broader and more integrative understanding of how social support may be associated with HIV-related outcomes. For example, prior research suggests that social support might not reduce sexual risk behaviour but it might reduce depression [23]. If depression is linked to risky sexual practices, we can target risky sex indirectly by addressing depression. Focusing on many outcomes in the same sample can allow one to develop a more comprehensive account of the implications of social support for health-related functioning.

To address some of the existing gaps in knowledge, this study aimed to contribute to the existing literature by assessing the associations between different types of social support and the sexual practices, health behaviour, and health outcomes of young women residing in an HIV endemic community in KwaZulu-Natal, South Africa. We hypothesized that tangible (cash and physical goods), educational, and emotional support in combination would generally show the strongest associations with the HIV risk–related outcomes, but we anticipated some variation in the pattern of associations by category of support and type of outcome.

## Methods

### Study Sample

This study is a secondary analysis of the data from the HIV Incidence Provincial Surveillance System (HIPSS) project in two sub-districts (Vulindlela and Greater Edendale) in uMgungundlovu District, KwaZulu-Natal province in South Africa [24]. uMgungundlovu is a HIV hyperendemic district with an antenatal HIV prevalence of 44% in 2016 [25]. Vulindlela is considered largely rural and Greater Edendale is largely peri urban. Men and women aged 15–49 years were enrolled from June 2014 to June 2015 (2014 Survey) and HIV seronegative participants aged 15–35 years had a single follow-up visit from June 2016 to April 2017.

All eligible participants provided written informed consent. Legal minors provided assent and written consent was obtained from parents, guardians, or caregivers. Face-to-face interviews were conducted by trained fieldworkers. The survey battery consisted of sociodemographic items, health-related measures, and HIV-related measures. Venous blood samples were collected from all participants and tested for HIV antibodies. The protocol paper provides a detailed description of the survey [24].

The analytical sample for this study consists of HIV-negative women from the 2014 Survey who were between 18 and 29 years of age (i.e. emerging adults) [26]. We limited the analysis to emerging adult females because this group is one of the more vulnerable subpopulations within the context of Eastern and Southern Africa [27]. In our baseline data, HIV prevalence in 2014 was about 3% among 18-year-olds and close to 30% among the 29-year-olds, implying an average of about 1.5% higher prevalence per 1 year increase in age. The incidence rate per 100 person-years was 4.00 for 20–24-year-olds and 2.29 for 25–29-year-olds [25].

The total sample consists of 1446 women, but the number of observations varies across the models estimated. The tables in the Electronic Supplementary Material contain information on the size of the sample used in each regression.

### Measures

#### Social Support

At baseline, participants indicated if they received (1) tangible support, such as money and food; (2) educational or informational support; and (3) emotional or relational support (bonding) from family, friends, work or organizations in the past month (Table 1 in Electronic Supplementary Material reports the frequencies). We used responses to these three forms of social support to create eight categories reflecting mutually exclusive combinations of social support: (1) no social support; (2) tangible support; (3) educational support; (4) emotional support; (5) educational + emotional support; (6) tangible + educational support; (7) tangible + emotional support; and (8) tangible + educational + emotional support.

**Table 1:** *Distribution of* b*aseline* p*articipant* c*haracteristics by* t*ype of* s*ocial* s*upport*

Characteristic	Total sample^a^ (*n* = 1021–1446)	Type of social support					
		Tangible		Educational		Emotional	
		No (*n* = 181–293)	Yes (*n* = 833–1153)	No (*n* = 541–754)	Yes (*n* = 517–875)	No (*n* = 418–611)	Yes (*n* = 596–835)
Sociodemographics							
Age (yrs), mean (*SD*)	22.3 (3.4)	23.0 (3.6)	22.2 (3.4)	22.73 (3.5)	21.5 (3.8)	22.5 (4)	22.2 (3.4)
Single, %	93.15	92.49	93.32	90.97	95.52	92.96	93.29
Education, %							
No schooling/crèche/pre-primary	1.95	7.17	0.61	3.32	0.44	1.64	2.17
Primary (grade 1–7)	1.88	2.73	1.66	2.26	1.46	1.81	1.93
Incomplete secondary (grade 8–11/NTC1/NTC2)	34.47	29.35	35.78	38.16	30.42	40.07	30.36
Completed secondary (grade 12/NTC3)	53.37	53.92	53.23	50.40	56.62	49.10	56.51
Tertiary (diploma or degree)	8.34	6.83	8.73	5.85	11.06	7.39	9.04
Away from home, %	9.63	7.88	10.08	9.44	9.84	8.55	10.42
Size of meals cut last 5 days, %	8.44	5.12	9.28	11.27	5.35	9.82	7.43
Household receives government grant	69.50	60.07	71.90	73.21	65.46	72.67	67.19
Wealth index quintiles, %							
Poorest	25.43	31.85	23.81	35.64	14.33	37.44	16.67
Poorer	15.45	18.49	14.68	19.15	11.43	19.05	12.83
Middle	19.40	19.86	19.29	17.95	20.98	17.41	20.86
Richer	20.24	18.84	20.59	15.82	25.04	15.27	23.86
Richest	19.47	10.96	21.63	11.44	28.22	10.84	25.78
Sexual practices							
More than one sex partner in last 12 months, %	8.28	17.13	6.36	8.31	8.24	10.29	6.88
Consistent condom use in last 12 months, %	20.67	19.91	20.86	20.48	20.90	20.21	21.02
Sex after alcohol in last 12 months, %	3.80	5.80	3.29	4.77	2.75	4.58	3.23
Sex after drug use in last 6 months, %	2.90	3.75	2.69	3.44	2.32	3.93	2.16
Health behaviour							
Alcohol use in last 12 months, %	9.19	10.58	8.85	10.08	8.23	9.98	8.62
Drug use in last 6 months, %	2.21	2.73	2.08	3.05	1.30	2.95	1.67
HIV test in last 12 months, %	21.99	37.54	18.04	21.08	22.97	25.20	19.64
Health							
Depression, %	27.17	52.90	20.64	23.87	30.78	26.18	27.90
HIV positive, %	-	-	-	-	-	-	-
Sexually transmitted infection (measured) in last 12 months, %	55.83	59.04	55.10	59.59	52.35	56.56	55.32

^a^Sample sizes within each category may vary because of missing data

#### Outcomes

Ten outcomes measured at baseline and follow-up were analysed, divided into sexual practices and risk behaviour (more than one sex partner, consistent condom use, sex after alcohol use, sex after drug use); health behaviour (alcohol use, drug use, HIV tested); and health (depression, HIV positive, self-reported sexually transmitted infection).

Depression was measured with the 10-item Center for Epidemiological Studies Depression Scale (CES-D10) [28]. The CES-D10 is a screening instrument that evaluates the degree of depressive symptoms experienced in the past week (e.g. “I felt depressed”). Two of the items are reverse scored, with total scores ranging from 0 to 30. The factorial and cross-cultural validity of the CES-D10 has been supported in a variety of subpopulations and languages [29–31], including those of South Africa [32]. Previous studies have typically used cut-off points on the CES-D10 at 8, 10, or 12 to identify individuals at risk for depression [31–33]. Our primary analysis uses a cut-off score of 8 to classify participants into low (< 8) and high (≥ 8) risk for depression groups, which corresponds with a cut-off score of 16 on the 20-item index [34]. In the present study, estimated internal consistency of the CES-D10 at baseline was *α* = 0.86.

HIV infection was measured with venous blood samples collected from all participants and tested for HIV antibodies with the Biomérieux Vironostika Uniform II Antigen/Antibody Microelisa system (BioMérieux, Marcy I’Etoile, France) and HIV 1/2 Combi Roche Elecsys (Roche Diagnostics, Penzberg, Germany). Positive tests were confirmed with a HIV-1 Western-blot assay (Biorad assay, Bio-Rad Laboratories, Redmond, WA 98052, USA).

At baseline, STIs (excluding HIV) were also measured with blood and self-collected genital samples, testing for *Neisseria gonorrhoeae*, *Chlamydia trachomatis*, *Trichomonas vaginalis* and *Mycoplasma genitalium*, syphilis, and genital herpes (HSV-2). In the follow-up survey, only self-reported information about STIs during the last 12 months was assessed. Results for STI are based on blood and self-collected genital samples at baseline and self-reported STI in the follow-up survey. However, we also estimated models using baseline values of self-reported STI, as well as of STI based on blood and self-collected genital samples without HSV-2, as one cannot recover from HSV-2, but the differences between those results were negligible.

All other outcomes are self-reported and dichotomous (0 = No; 1 = Yes). Survey items on number of sex partners’ consistent condom use, had taken a HIV test (excluding the one taken at the 2014 Survey), used alcohol, and had sex after alcohol use refer to the past 12 months, whereas questions about drug use (dagga, heroin, cocaine, glue, tik, wunga, etc.) and sex after drug use refer to the past 6 months. The variable “number of sex partners” assesses whether participants had more than one sexual partner in the past 12 months, so all outcomes are dichotomous with 0 = No and 1 = Yes.

#### Covariates

To control for heterogeneity in the sample, all models were adjusted for age dichotomized into 3-year age groups (e.g. 18–20, 21–23), educational attainment (no schooling or crèche/pre-primary, primary, incomplete secondary, completed secondary, tertiary education), household wealth (quantiles based on an index of physical assets), government grants received by household (no grant or at least one grant out of eight available grants), single (not married or in union), having reduced meals during the last 5 days (a measure of poverty), and spent more than a month away from home in the previous year.

### Data Analyses

All analyses were conducted using Stata 16. In the primary analysis, we regressed each outcome assessed in the follow-up wave (2016/2017) on social support assessed at baseline (2014/2015), adjusting for covariates assessed at baseline. Except for the HIV status outcome (which did not have any variability at baseline because all participants in the analytic sample were HIV negative), each model controlled for prior values of the respective outcome variable assessed at baseline. An available-case analytic approach was used. Consistent with recent recommendations [35] and prior studies [36, 37], results tables report statistical significance both before and after Bonferroni correction. Weights, adjusted for nonresponse at baseline and follow-up, were included to account for the probability of selecting the enumeration area, the household in the enumeration area, and the individual in the household (for details, see [38]).

The “no social support” category was used as the reference group, with effect estimates indicating the associations between each of the seven social support categories and the outcomes relative to the reference group in the analysis for the adjusted odds ratios. We used *E*-values to evaluate the sensitivity of the estimated effects to unmeasured confounding [39].

## Results

The sociodemographic characteristics of the participants, both in the full analytic sample and stratified by forms of social support, are reported in Table 1. The average age of the sample was 22.3 years, over 93% of participants were not in a relationship, and a majority had completed secondary education or higher (62%). The distribution of wealth was roughly equal across the quintiles, but there are more participants in the lowest quintile (25%) than in the highest (19%). About 8% of the sample had reduced the size of their meals during the last 5 days, close to 70% lived in households that received at least one government grant, and 10% had been away from home for more than a month during the last 12 months.

Table 2 reports logistic regression model estimates for the seven combinations of social support and the ten outcomes (see Electronic Supplementary Material Tables 2 to 5 for complete results). Tangible + emotional support was associated with lower odds of more than one sex partner (adjusted odds ratio [AOR] = 0.22, *p* = 0.0036), sex after alcohol use (AOR = 0.37, *p* = 0.0009), alcohol use (AOR = 0.32, *p* = 0.0001), and depression (AOR = 0.43, *p* = 0.0047). Similarly, all three forms of support in combination were associated with lower odds of sex after alcohol use (AOR = 0.31, *p* = 0.0010), alcohol use (AOR = 0.30, *p* = 0.0004), and drug use (AOR = 0.23, *p* = 0.0035). All three forms of support in combination were associated with lower odds of subsequently having more than one sex partner (AOR = 0.27, *p* = 0.0084).

**Table 2:** Associations of social support (2014 Survey) with subsequent, sexual practices, health behaviour, and health)^a^

Outcome	Social support						
	Tangible	Educational	Emotional	Educational and emotional	Tangible and educational	Tangible and emotional	Tangible, educational, and emotional
Sexual practices							
More than one sex partner	0.29* [0.11, 0.76]	0.15 [0.02, 1.55]	0.53 [0.10, 2.65]	0.55 [0.07, 4.14]	0.82 [0.20, 3.38]	0.22** [0.08, 0.60]	0.27* [0.11, 0.72]
Consistent condom use	0.79 [0.41, 1.53]	0.23 [0.04, 1.33]	0.87 [0.16, 4.66]	0.50 [0.14, 1.78]	0.38 [0.08, 1.74]	1.13 [0.48, 2.63]	0.73 [0.38, 1.39]
Sex after alcohol use	0.44* [0.23, 0.85]	0.90 [0.29, 2.81]	0.83 [0.24, 2.90]	0.87 [0.36, 2.07]	0.73 [0.29, 1.79]	0.37** [0.20, 0.66]	0.31** [0.15, 0.62]
Sex after drug use	0.20* [0.05, 0.77]	0.67 [0.10, 4.68]	-	1.16 [0.21, 6.41]	0.13 [0.00, 8.47]	0.14 [0.01, 1.41]	0.19* [0.04, 0.84]
Health behaviour							
Alcohol use	0.44* [0.23, 0.82]	0.97 [0.33, 2.87]	0.72 [0.22, 2.31]	0.94 [0.43, 2.09]	0.68 [0.28, 1.64]	0.32** [0.18, 0.57]	0.30** [0.15, 0.58]
Drug use	0.19* [0.04, 0.82]	0.60 [0.05, 6.67]	2.75 [0.33, 22.94]	1.05 [0.19, 5.86]	0.72 [0.16, 3.25]	0.33 [0.08, 1.27]	0.23** [0.09, 0.62]
HIV tested	0.89 [0.44, 1.82]	0.47 [0.12, 1.84]	0.99 [0.30, 3.30]	1.24 [0.46, 3.38]	1.75 [0.68, 4.50]	1.06 [0.51, 2.19]	1.30 [0.67, 2.51]
Health							
Depression	0.48** [0.29, 0.80]	1.18 [0.39, 3.55]	1.52 [0.60, 3.84]	0.71 [0.29, 1.72]	0.46 [0.19, 1.07]	0.43** [0.24, 0.77]	0.57* [0.33, 0.99]
HIV status	0.65 [0.25, 1.73]	-	1.85 [0.36, 9.50]	1.14 [0.26, 4.99]	1.72 [0.45, 6.60]	0.52 [0.15, 1.81]	0.80 [0.30, 2.14]
Sexually transmitted infection (measured at baseline, self-reported at follow-up)	1.08 [0.32, 3.65]	0.43 [0.04, 4.90]	0.73 [0.18, 2.96]	1.13 [0.20, 6.35]	1.08 [0.16, 7.09]	0.99 [0.21, 4.65]	0.71 [0.20, 2.45]

^*^p < .05 before but not after Bonferroni correction; **p < .05 after Bonferroni correction (the *p*-value cut-off for Bonferroni correction was .05/10 = .005 for each outcome). Some estimates are missing because of few observations

^a^Estimated odds ratios and 95% confidence intervals in brackets. An outcome-wide analytic approach was used to estimate effects, which involved regressing each outcome on tangible, educational, and emotional support. The number of observations in the models ranges from 953 to 1700. Logistic regressions were used to estimate the odds ratios. All models are adjusted for prior values of 3-year age dummies, educational attainment, household assets, marital status, size of the meal cut last 5 days, away from home for more than a month during the last 12 months, and number of government grants received, assessed in in the baseline survey. Each model also controls for prior values of the respective outcomes assessed in the baseline survey, except HIV positive since all women in the follow-up survey were HIV negative. Educational support for HIV status and emotional support for sex after drugs predicted failure perfectly so they were omitted

Tangible support alone and all three forms of support in combination evidenced a more modest association compared to the results above, with lower odds of more than one sex partner, sex after alcohol use, sex after drug use, and depression (AORs = 0.20 to 0.57, *ps* ≤ 0.0500). There was little evidence to suggest that emotional and educational social support was independently associated with any of the abovementioned outcomes (*ps* > 0.05).

We did not find any evidence of associations between social support and subsequent HIV infection, STI status, consistent condom use, and tested for HIV (*ps* > 0.05). It is likely that the findings for HIV infection, consistent condom use, and tested for HIV are due to the exclusion of women who were HIV-positive, while the finding for STI status is more likely to be due to measurement errors in the follow-up survey. Nevertheless, we re-estimated the models for the four outcomes with teenagers (i.e. 15–19-year-olds), who should be less likely to have been infected by HIV (see Table 6 in the Electronic Supplementary Material). Providing some evidence that this might be the case, we found that tangible support was associated with lower odds of HIV infection (AOR = 0.20, *p* = 0.0460). We did not find any other evidence of associations in this subsample, but all AORs were in the expected direction. Given the small number of observations in this subsample (*n* = 227–654), these analyses may not have been sufficiently powered.

The choice of cut-off for the depression variable varies somewhat across studies [40]. Thus, as a robustness check, we report estimates with cut-offs at 10 and 12 (see Table 7 in the Electronic Supplementary Material). The results for cut-offs of 10 and 12 are somewhat weaker than those obtained with a cut-off of at 8, but the AORs for tangible support are similar and significantly excluded the null (AORs = 0.53, *p* = 0.0237; AORs = 0.43, *p* = 0.0045).

Since there are few observations in some categories, we re-estimated the models with a condensed measure of support. Table 8 in the Electronic Supplementary Material reports estimates of models with three categories, no support (reference category), tangible support only or combined with educational and/or emotional support, and educational and/or emotional support. The results suggest that emotional and educational support has more limited implications for the outcomes when they are not combined with tangible support.

*E*-values for the sensitivity analysis corresponding with the primary analysis are reported in Table 3. *E*-values for the effect estimates across the types of social support ranged from 1.06 to 15.07, suggesting that some of the results were at least moderately robust to potential unmeasured confounding. Slightly lower *E*-values were found for the limit of the confidence interval (range: 1.00, 2.92). There were variations in strength of *E*-values by type of support. For example, *E*-values for the effect estimates associated with educational and emotional support varied from 1.28 to 3.43, whereas the range for the *E*-values of the effect estimates of tangible support ranged more widely from 1.30 to 9.94.

**Table 3:** *Robustness to* u*nmeasured* c*onfounding (E-values*^*a*^*) for the* a*ssociations of* s*ocial* s*upport at* b*aseline with* s*ubsequent sexual practices, health,* and *health* *behaviour, * s*exual* *practices and* *risk* *behaviour*

Outcome	Exposure						
	Tangible	Educational	Emotional	Educational and emotional	Tangible and educational	Tangible and emotional	Tangible, educational, and emotional
Sexual practices							
More than one sex partner							
Effect estimate^b^	6.29	12.42	3.20	3.02	1.72	8.65	6.75
Confidence interval limit^c^	1.94	1.00	1.00	1.00	1.00	2.70	2.14
Consistent condom use							
Effect estimate^b^	1.85	8.19	1.56	3.43	4.70	1.50	2.10
Confidence interval limit^c^	1.00	1.00	1.00	1.00	1.00	1.00	1.00
Sex after alcohol use							
Effect estimate^b^	4.00	1.46	1.70	1.57	2.10	4.91	6.00
Confidence interval limit^c^	1.65	1.00	1.00	1.00	1.00	2.40	2.62
Sex after drug use							
Effect estimate^b^	9.24	2.33	-	1.59	15.07	13.72	10.08
Confidence interval limit^c^	1.93	1.00	-	1.00	1.00	1.00	1.65
Health behaviour							
Alcohol use							
Effect estimate^b^	3.99	1.19	2.14	1.31	2.29	5.74	6.15
Confidence interval limit^c^	1.74	1.00	1.00	1.00	1.00	2.92	2.86
Drug use							
Effect estimate^b^	9.94	2.70	4.94	1.28	2.11	5.56	8.00
Confidence interval limit^c^	1.72	1.00	1.00	1.00	1.00	1.00	2.63
HIV tested							
Effect estimate^b^	1.30	2.27	1.06	1.47	1.98	1.20	1.54
Confidence interval limit^c^	1.00	1.00	1.00	1.00	1.00	1.00	1.00
Health							
Depression							
Effect estimate^b^	2.24	1.39	1.76	1.67	2.32	2.42	1.99
Confidence interval limit^c^	1.48	1.00	1.00	1.00	1.00	1.54	1.09
HIV positive							
Effect estimate^b^	3.54	-	3.80	1.61	2.44	3.21	1.92
Confidence interval limit^c^	1.00	-	1.00	1.00	1.00	1.00	1.00
Sexually transmitted infection							
Effect estimate^b^	1.37	4.10	2.09	1.52	1.36	1.09	2.18
Confidence interval limit^c^	1.00	1.00	1.00	1.00	1.00	1.00	1.00

^a^The formula for calculating *E*-values can be found in VanderWeele and Ding [39]

^b^*E*-values for effect estimates are the minimum strength of association that an unmeasured confounder would need to have with both the exposure and the outcome to fully explain away the observed effect, after accounting for the measured covariates

^c^*E*-values for the limit of the 95% confidence interval closest to the null denote the minimum strength of association that an unmeasured confounder would need to have with both the exposure and the outcome to shift the confidence interval to include the null value, after accounting for the measured covariates

## Discussion

Our findings suggest that social support has the potential to effectively reduce various risk factors related to HIV infection among emerging adult women in South Africa. A key finding is that tangible social support was associated with a lower likelihood of more than one sexual partner, sex after alcohol and drug use, use of alcohol and drugs, and depression. This pattern of findings suggests that a lack of money could play an important role in the sexual and health behaviours of young women. In contexts where poverty is high, the ability to purchase various basic necessities might reduce psychosocial problems and willingness to engage in transactional sex (i.e. a sexual relationship where receiving gifts, money, or other services is an important factor in a person’s decision to engage in sex) [41].

Several of the strongest associations were observed when tangible support and emotional and educational support were combined at baseline, demonstrating the potential benefit of combinations of support in mitigating HIV risk outcomes. This might be because tangible support strengthens the effects of emotional and educational support. We also found little evidence to suggest that educational and emotional support was independently associated with the outcomes included in this study. One possible reason for this finding is that education and emotional support might be less relevant to emerging adult women living in contexts of poverty.

The findings of the study are subject to limitations. The lack of evidence of associations between social support and certain HIV risk–related outcomes (e.g. HIV infection, STIs, consistent condom use, HIV testing) could be due the short period of time between the surveys (approximately 1 year). Although HIV and STI incidence rates are high in the study area according to most standards, the number of new infections is still small. Therefore, a longer lag between waves may be needed for more definitive evidence of associations to be observed. However, underreporting is also a likely reason for not finding an association with STI. For consistent condom use, it is possible that this outcome depends more on whether a partner can be trusted than on a plan that can be affected by support from parents or others. After all, only approximately 20% of the girls and women used condoms consistently with their last partner in the two surveys, and only 7% reported using condoms consistently in both surveys. Another potential explanation for the lack of evidence of associations between social support and HIV infection, STIs, consistent condom use, or HIV testing is the exclusion of many women at high risk of being infected with HIV, since the follow-up survey only included women who were HIV-negative at baseline. That is, women who were not using condoms consistently were at higher risk of being infected by HIV and STIs than others, and they might have been more likely to have had a (positive) HIV test. To provide some evidence for this argument, models were re-estimated for adolescent girls in the sample (i.e. 15–19-year-olds), since they are less likely to have been infected by HIV than older women, even if they engaged in risky sex. Although not definitive, some of the results indicated that tangible support might have reduced the risk of HIV infection among the adolescent girls.

Another limitation is that most of the outcomes included in the present study are negative factors, but social support may be more strongly associated with positive outcomes (e.g. better performance in school, greater happiness). Moreover, we used general measures of social support that differentiated between broad categories of support, but they do not capture the nuances of the social support that participants received (e.g. amount of support, frequency of support). Finally, given that HIPSS is limited to two waves of data and not collected with the aim of testing theories, we were unable to appropriately evaluate theoretically informed mechanisms that might explain some of our findings. Further research is needed to explore the relative contribution of different theoretical perspectives to understanding how different combinations of social support might be related to HIV risk–related outcomes among emerging adult women.

In conclusion, our findings add to recent research that has evaluated the effects of cash transfer programmes and the synergistic effects of combining them with caregiver care [16, 19]. Many existing poverty and HIV interventions focus on tangible support (e.g. cash transfers to households), possibly with some conditionality attached to the provision of such resources, or on parenting education to improve their children’s behaviour and well-being [20, 42]. Although our findings suggest that tangible support may play a key role in mitigating HIV risk–related outcomes, they also indicate that it may be prudent to combine cash transfers with psychosocial care and parenting education when designing HIV prevention programmes for emerging adult women in high HIV burdened contexts.

### Supplementary Information

Below is the link to the electronic supplementary material.

Supplementary file1 (DOCX 75 KB)

## Data Availability

The datasets used and or analysed during the current study are available from the corresponding author on reasonable request.
