# Supplementary material for: Associations of Social Support with Sexual Practices, Health Behaviours, and Health Outcomes Among Adolescent Girls and Young Women: Evidence From a Longitudinal Study in KwaZulu-Natal, South Africa
Source: Int J Behav Med. 2023 Jul 21;31(4):620–30. doi: 10.1007/s12529-023-10199-6 (PMC11269318; doi:10.1007/s12529-023-10199-6)
Supplement: Supplementary file 1 — Supplementary file1 (DOCX 75 KB) [file 12529_2023_10199_MOESM1_ESM.docx]

# Electronic Supplementary Material

**Social support**

**Table 1** Distribution of social support, women 18-29 years.

|  | Tangible | | Educational | | Emotional | |
| --- | --- | --- | --- | --- | --- | --- |
|  | Frequency | Percent | Frequency | Percent | Frequency | Percent |
| Yes | 1153 | 80 | 692 | 48 | 835 | 58 |
| No | 293 | 20 | 754 | 52 | 611 | 42 |
| Total | 1446 | 100 | 1446 | 100 | 1446 | 100 |

**Tables 2-5, the main regressions**

**Table 2** Social support, women 18-29 years. Adjusted odds ratios and 95% confidence intervals.

|  |  |  |
| --- | --- | --- |
| VARIABLES | More than one sex partner last 12 months | Consistent condom use last 12 months |
|  |  |  |
| **Social support** |  |  |
| Tangible | 0.293* | 0.789 |
|  | (0.112 - 0.765) | (0.406 - 1.534) |
| Educational | 0.155 | 0.229 |
|  | (0.015 - 1.548) | (0.040 - 1.330) |
| Emotional | 0.527 | 0.870 |
|  | (0.105 - 2.651) | (0.162 - 4.662) |
| Educational and Emotional | 0.553 | 0.498 |
|  | (0.074 - 4.141) | (0.140 - 1.779) |
| Tangible and Educational | 0.824 | 0.381 |
|  | (0.201 - 3.377) | (0.083 - 1.735) |
| Tangible and Emotional | 0.218** | 1.125 |
|  | (0.079 - 0.604) | (0.481 - 2.631) |
| All forms of support | 0.274** | 0.727 |
|  | (0.105 - 0.716) | (0.380 - 1.390) |
| More than one sex partner last 12 months at baseline | 3.936*** |  |
|  | (1.789 - 8.663) |  |
| Consistent condom use last 12 months at baseline |  | 2.362*** |
|  |  | (1.434 - 3.891) |
| **Age** |  |  |
| 21-23 years | 0.645 | 1.109 |
|  | (0.348 - 1.195) | (0.653 - 1.884) |
| 24-26 years | 0.655 | 0.889 |
|  | (0.315 - 1.362) | (0.503 - 1.569) |
| 27-29 years | 0.563 | 1.422 |
|  | (0.214 - 1.481) | (0.878 - 2.302) |
| **Education** |  |  |
| Primary | 0.433 | 1.628 |
|  | (0.043 - 4.342) | (0.154 - 17.184) |
| Incomplete secondary | 1.082 | 1.533 |
|  | (0.353 - 3.317) | (0.241 - 9.763) |
| Completed secondary | 0.872 | 1.945 |
|  | (0.305 - 2.489) | (0.298 - 12.705) |
| Tertiary | - | - |
|  |  |  |
| Single | 0.796 | 3.885** |
|  | (0.244 - 2.597) | (1.485 - 10.162) |
| Away from home | 1.060 | 0.485 |
|  | (0.417 - 2.694) | (0.225 - 1.046) |
| Size of meal cut last 5 days | 1.534 | 0.915 |
|  | (0.751 - 3.133) | (0.497 - 1.685) |
| **Wealth index quintile** |  |  |
| Poorer | 0.617 | 0.815 |
|  | (0.261 - 1.458) | (0.427 - 1.556) |
| Middle | 0.914 | 0.749 |
|  | (0.427 - 1.959) | (0.393 - 1.428) |
| Richer | 0.797 | 1.000 |
|  | (0.316 - 2.006) | (0.545 - 1.836) |
| Richest | 2.230 | 1.023 |
|  | (0.927 - 5.366) | (0.505 - 2.071) |
| Government grant | 0.821 | 1.041 |
|  | (0.456 - 1.479) | (0.704 - 1.540) |
| Constant | 0.380 | 0.044** |
|  | (0.069 - 2.090) | (0.004 - 0.450) |
|  |  |  |
| Observations | 903 | 1,015 |

*Note*. **p* < 0.05 ***p* < 0.01 ****p* < 0.001. All outcome variables are dichotomous. Weights are used to account for the probability of selecting the enumeration area, the household in the enumeration area, and the individual in the household, adjusted for nonresponse.

**Table 3** Social support, women 18-29 years. Adjusted odds ratios and 95% confidence intervals.

|  |  |  |
| --- | --- | --- |
| VARIABLES | Sex after drinking last 12 months | Sex after drugs last 12 months |
|  |  |  |
| **Social support** |  |  |
| Tangible | 0.437* | 0.205* |
|  | (0.226 - 0.845) | (0.055 - 0.768) |
| Educational | 0.901 | 0.674 |
|  | (0.289 - 2.812) | (0.097 - 4.678) |
| Emotional | 0.831 |  |
|  | (0.239 - 2.897) |  |
| Educational and Emotional | 0.868 | 1.158 |
|  | (0.365 - 2.066) | (0.209 - 6.409) |
| Tangible and Educational | 0.726 | 0.128 |
|  | (0.295 - 1.786) | (0.002 - 8.468) |
| Tangible and Emotional | 0.366*** | 0.141 |
|  | (0.203 - 0.660) | (0.014 - 1.415) |
| All forms of support | 0.306** | 0.189* |
|  | (0.151 - 0.617) | (0.042 - 0.844) |
| Sex after drinking last 12 months at baseline | 2.862** |  |
|  | (1.421 - 5.766) |  |
| Sex after drugs last 6 months at baseline |  | 45.462*** |
|  |  | (9.139 - 226.150) |
| **Age** |  |  |
| 21-23 years | 1.258 | 0.165 |
|  | (0.860 - 1.838) | (0.019 - 1.412) |
| 24-26 years | 0.757 | 1.096 |
|  | (0.459 - 1.249) | (0.238 - 5.054) |
| 27-29 years | 0.864 | 0.235 |
|  | (0.516 - 1.448) | (0.034 - 1.610) |
| **Education** |  |  |
| Primary | 1.081 | 11.259* |
|  | (0.129 - 9.042) | (1.625 - 78.002) |
| Incomplete secondary | 0.725 | 1.340 |
|  | (0.188 - 2.797) | (0.323 - 5.563) |
| Completed secondary | 0.881 |  |
|  | (0.233 - 3.325) |  |
| Tertiary | 1.064 |  |
|  | (0.252 - 4.495) |  |
| Single | 1.790 | 0.280 |
|  | (0.797 - 4.024) | (0.063 - 1.257) |
| Away from home | 0.562 | 0.343 |
|  | (0.293 - 1.075) | (0.033 - 3.580) |
| Size of meal cut last 5 days | 1.294 | 0.806 |
|  | (0.673 - 2.488) | (0.133 - 4.869) |
| **Wealth index quintile** |  |  |
| Poorer | 0.657 | 0.604 |
|  | (0.366 - 1.180) | (0.099 - 3.684) |
| Middle | 0.896 | 1.490 |
|  | (0.485 - 1.653) | (0.326 - 6.809) |
| Richer | 0.743 | 0.247 |
|  | (0.382 - 1.445) | (0.033 - 1.848) |
| Richest | 1.102 | 0.926 |
|  | (0.624 - 1.945) | (0.165 - 5.207) |
| Government grant | 1.112 | 0.452 |
|  | (0.751 - 1.645) | (0.116 - 1.765) |
| Constant | 0.246 | 0.130 |
|  | (0.046 - 1.319) | (0.010 - 1.704) |
|  |  |  |
| Observations | 1,432 | 1,257 |

*Note*. **p* < 0.05 ***p* < 0.01 ****p* < 0.001. All outcome variables are dichotomous. Weights are used to account for the probability of selecting the enumeration area, the household in the enumeration area, and the individual in the household, adjusted for nonresponse.

**Table 4** Social support, women 18-29 years. Adjusted odds ratios and 95% confidence intervals.

|  |  |  |  |
| --- | --- | --- | --- |
| VARIABLES | Alcohol use last 12 months | Drug use last 12 months | HIV tested last 12 months |
|  |  |  |  |
| **Social support** |  |  |  |
| Tangible | 0.439* | 0.191* | 0.894 |
|  | (0.235 - 0.820) | (0.044 - 0.824) | (0.439 - 1.821) |
| Educationalal | 0.974 | 0.603 | 0.470 |
|  | (0.331 - 2.865) | (0.055 - 6.666) | (0.120 - 1.838) |
| Emotional | 0.715 | 2.747 | 0.994 |
|  | (0.221 - 2.313) | (0.329 - 22.944) | (0.300 - 3.296) |
| Educational and Emotional | 0.945 | 1.051 | 1.243 |
|  | (0.427 - 2.089) | (0.188 - 5.863) | (0.457 - 3.382) |
| Tangible and Educational | 0.683 | 0.723 | 1.750 |
|  | (0.284 - 1.644) | (0.160 - 3.254) | (0.681 - 4.499) |
| Tangible and Emotional | 0.318*** | 0.327 | 1.057 |
|  | (0.178 - 0.568) | (0.085 - 1.266) | (0.509 - 2.195) |
| All forms of support | 0.299*** | 0.235** | 1.297 |
|  | (0.155 - 0.577) | (0.089 - 0.617) | (0.670 - 2.509) |
| Alcohol use last 12 months at baseline | 4.032*** |  |  |
|  | (2.505 - 6.490) |  |  |
| Drugs use last 6 months at baseline |  | 23.720*** |  |
|  |  | (6.029 - 93.320) |  |
| HIV tested at baseline |  |  | 1.721* |
|  |  |  | (1.097 - 2.699) |
| **Age** |  |  |  |
| 21-23 years | 1.243 | 1.056 | 0.897 |
|  | (0.835 - 1.851) | (0.376 - 2.967) | (0.582 - 1.381) |
| 24-26 years | 0.801 | 0.800 | 0.729 |
|  | (0.497 - 1.291) | (0.234 - 2.735) | (0.405 - 1.311) |
| 27-29 years | 0.942 | 0.451 | 0.913 |
|  | (0.555 - 1.599) | (0.091 - 2.233) | (0.517 - 1.613) |
| **Education** |  |  |  |
| Primary | 1.394 | 28.130 | 2.808 |
|  | (0.184 - 10.571) | (0.517 - 1,530.779) | (0.487 - 16.201) |
| Incomplete secondary | 0.818 | 10.195 | 2.025 |
|  | (0.215 - 3.109) | (0.218 - 477.530) | (0.576 - 7.122) |
| Completed secondary | 0.840 | 3.736 | 1.295 |
|  | (0.227 - 3.107) | (0.091 - 152.873) | (0.368 - 4.561) |
| Tertiary | 0.981 |  | 0.774 |
|  | (0.233 - 4.140) |  | (0.180 - 3.327) |
| Single | 1.750 | 0.747 | 1.441 |
|  | (0.784 - 3.903) | (0.169 - 3.311) | (0.586 - 3.540) |
| Away from home | 0.555 | 0.525 | 1.029 |
|  | (0.291 - 1.059) | (0.092 - 3.005) | (0.575 - 1.841) |
| Size of meal cut last 5 days | 1.469 | 1.803 | 1.074 |
|  | (0.801 - 2.693) | (0.672 - 4.841) | (0.528 - 2.181) |
| **Wealth index quintile** |  |  |  |
| Poorer | 0.639 | 1.538 | 0.891 |
|  | (0.360 - 1.133) | (0.428 - 5.530) | (0.458 - 1.733) |
| Middle | 0.912 | 1.680 | 0.998 |
|  | (0.511 - 1.630) | (0.507 - 5.566) | (0.548 - 1.817) |
| Richer | 0.728 | 0.730 | 0.889 |
|  | (0.380 - 1.395) | (0.140 - 3.810) | (0.491 - 1.608) |
| Richest | 1.113 | 0.935 | 0.863 |
|  | (0.635 - 1.949) | (0.244 - 3.579) | (0.475 - 1.566) |
| Government grant | 0.981 | 0.581 | 1.170 |
|  | (0.659 - 1.460) | (0.217 - 1.556) | (0.741 - 1.846) |
|  |  |  | (1.413 - 2.900) |
| Constant | 0.269 | 0.010* | 0.087** |
|  | (0.053 - 1.370) | (0.000 - 0.638) | (0.017 - 0.440) |
|  |  |  |  |
| Observations | 1,432 | 1,311 | 1,432 |

*Note*. **p* < 0.05 ***p* < 0.01 ****p* < 0.001. All outcome variables are dichotomous. Weights are used to account for the probability of selecting the enumeration area, the household in the enumeration area, and the individual in the household, adjusted for nonresponse.

**Table 5** Social support, women 18-29. Adjusted odds ratios and 95% confidence intervals.

|  |  |  |  |
| --- | --- | --- | --- |
| VARIABLES | Depression | HIV status | STI last 12 months |
|  |  |  |  |
| **Social support** |  |  |  |
| Tangible | 0.480** | 0.652 | 1.078 |
|  | (0.288 - 0.799) | (0.246 - 1.730) | (0.318 - 3.654) |
| Educational | 1.179 |  | 0.428 |
|  | (0.391 - 3.555) |  | (0.037 - 4.895) |
| Emotional | 1.515 | 1.848 | 0.727 |
|  | (0.598 - 3.837) | (0.359 - 9.504) | (0.179 - 2.960) |
| Education and Emotional | 0.706 | 1.139 | 1.133 |
|  | (0.290 - 1.718) | (0.260 - 4.990) | (0.202 - 6.348) |
| Tangible and Educational | 0.457 | 1.715 | 1.076 |
|  | (0.194 - 1.074) | (0.445 - 6.605) | (0.163 - 7.092) |
| Tangible and Emotional | 0.430** | 0.524 | 0.994 |
|  | (0.240 - 0.769) | (0.152 - 1.808) | (0.212 - 4.650) |
| All forms of support | 0.568* | 0.804 | 0.706 |
|  | (0.326 - 0.988) | (0.302 - 2.143) | (0.203 - 2.452) |
| Depression at baseline_ | 0.752 |  |  |
|  | (0.532 - 1.063) |  |  |
| STI at baseline |  |  | 1.576 |
|  |  |  | (0.807 - 3.080) |
| **Age** |  |  |  |
| 21-23 years | 1.266 | 0.815 | 1.835 |
|  | (0.913 - 1.756) | (0.437 - 1.519) | (0.872 - 3.862) |
| 24-26 years | 1.017 | 0.346** | 0.856 |
|  | (0.661 - 1.565) | (0.166 - 0.722) | (0.240 - 3.048) |
| 27-29 years | 1.102 | 0.551 |  |
|  | (0.700 - 1.733) | (0.245 - 1.235) |  |
| **Education** |  |  |  |
| Primary | 1.056 |  |  |
|  | (0.288 - 3.871) |  |  |
| Incomplete secondary | 1.187 | 1.081 | 0.268 |
|  | (0.452 - 3.115) | (0.178 - 6.568) | (0.068 - 1.052) |
| Completed secondary | 1.321 | 0.907 | 0.276 |
|  | (0.513 - 3.398) | (0.151 - 5.427) | (0.068 - 1.117) |
| Tertiary | 1.347 | 0.542 | 0.200 |
|  | 1.056 |  |  |
| Single | 0.854 | 6.166 | 0.807 |
|  | (0.512 - 1.425) | (0.745 - 51.034) | (0.098 - 6.661) |
| Away from home | 0.581 | 1.108 | 3.419* |
|  | (0.337 - 1.003) | (0.533 - 2.301) | (1.318 - 8.867) |
| Size of meal cut last 5 days | 0.747 | 1.349 | 0.884 |
|  | (0.490 - 1.138) | (0.639 - 2.849) | (0.292 - 2.679) |
| **Wealth index quintile** |  |  |  |
| Poorer | 0.708 | 0.874 | 1.139 |
|  | (0.458 - 1.094) | (0.438 - 1.745) | (0.402 - 3.228) |
| Middle | 1.099 | 0.676 | 0.853 |
|  | (0.704 - 1.715) | (0.286 - 1.598) | (0.307 - 2.369) |
| Richer | 0.862 | 0.480 | 0.841 |
|  | (0.556 - 1.336) | (0.174 - 1.323) | (0.291 - 2.425) |
| Richest | 0.980 | 0.404* | 1.055 |
|  | (0.608 - 1.580) | (0.176 - 0.930) | (0.420 - 2.650) |
| Government grant | 1.074 | 1.012 | 0.715 |
|  | (0.770 - 1.498) | (0.557 - 1.839) | (0.360 - 1.421) |
| Constant | 1.713 | 0.030* | 0.189 |
|  | (0.538 - 5.451) | (0.002 - 0.488) | (0.017 - 2.050) |
|  |  |  |  |
| Observations | 1,432 | 1,375 | 997 |

*Note*. **p* < 0.05 ***p* < 0.01 ****p* < 0.001. All outcome variables are dichotomous. Weights are used to account for the probability of selecting the enumeration area, the household in the enumeration area, and the individual in the household, adjusted for nonresponse.

**Additional regressions**

**Using a sample of teenagers (15-19 years)**

**Table 6** Social support, women 15-19 years. Adjusted odds ratios and 95% confidence intervals.

|  |  |  |  |  |
| --- | --- | --- | --- | --- |
| VARIABLES | HIV status | Consist condom use last 12 months | STI last 12 months | HIV tested last 12 months |
|  |  |  |  |  |
| **Social support** |  |  |  |  |
| Tangible | 0.195** | 1.547 | 0.189 | 1.523 |
|  | (0.039 - 0.969) | (0.122 - 19.66) | (0.0179 - 1.989) | (0.423 - 5.483) |
| Educational | - | - | - | 4.192 |
|  |  |  |  | (0.593 - 29.653) |
| Emotional | 1.697 | - | - | 1.163 |
|  | (0.174 - 16.543) |  |  | (0.172 - 7.863) |
| Educational and Emotional | 0.486 | 1.268 | 0.284 | 4.365* |
|  | (0.039 - 6.130) | (0.0322 - 49.89) | (0.0199 - 4.040) | (1.069 - 17.820) |
| Tangible and Educational | 0.538 | 1.904 |  | 4.390 |
|  | (0.084 - 3.427) | (0.172 - 21.06) |  | (0.939 - 20.515) |
| Tangible and Emotional | 0.231 | 1.846 | 0.175 | 1.179 |
|  | (0.027 - 1.952) | (0.153 - 22.24) | (0.0166 - 1.844) | (0.232 - 5.981) |
| All forms of support | 0.379 | 1.274 | 0.241 | 2.600 |
|  | (0.090 - 1.588) | (0.138 - 11.80) | (0.0448 - 1.296) | (0.765 - 8.837) |
| Consist condom use at baseline |  | 3.342* |  |  |
|  |  | (1.291 - 8.653) |  |  |
| STI at baseline |  |  | 1.865 |  |
|  |  |  | (0.796-4.363) |  |
| HIV tested at baseline |  |  |  | 2.412*** |
|  |  |  |  | (1.520 - 3.825) |
| **Age** |  |  |  |  |
| 18-19 years | 3.560*** | 0.815 | 1.765 | 0.536* |
|  | (1.549 - 8.182) | (0.285 - 2.334) | (0.763 - 4.081) | (0.326 - 0.883) |
| **Education** |  |  |  |  |
| Primary | 4.534 | 10.33 | 2.423 | 0.740 |
|  | (0.140 - 146.506) | (0.539 - 198.2) | (0.193 - 30.37) | (0.086 - 6.373) |
| Incomplete secondary | 1.905 | 2.097 | 0.275 | 0.792 |
|  | (0.115 - 31.601) | (0.272 - 16.18) | (0.0426 - 1.772) | (0.124 - 5.044) |
| Completed secondary | 1.914 | 1.901 | 0.250 | 0.627 |
|  | (0.106 - 34.417) | (0.235 - 15.41) | (0.0361 - 1.735) | (0.100 - 3.914) |
| Tertiary | 3.378 |  |  | 0.204 |
|  | (0.130 - 87.937) |  |  | (0.015 - 2.842) |
| Single |  | 0.706 |  | 0.282 |
|  |  | (0.0857 - 5.809) |  | (0.038 - 2.110) |
| Away from home | 0.848 | 1.449 | 1.519 | 2.014** |
|  | (0.297 - 2.418) | (0.271 - 7.743) | (0.378 - 6.102) | (1.017 - 3.988) |
| Size of meal cut last 5 days | 0.409 | 0.431 | 0.258 | 0.732 |
|  | (0.080 - 2.085) | (0.0657 - 2.832) | (0.0283 - 2.361) | (0.333 - 1.606) |
| **Wealth index quintile** |  |  |  |  |
| Poorer | 0.530 | 1.482 | 0.369 | 0.986 |
|  | (0.185 - 1.515) | (0.368 - 5.971) | (0.0674 - 2.018) | (0.469 - 2.075) |
| Middle | 0.448 | 0.556 | 0.902 | 0.843 |
|  | (0.167 - 1.200) | (0.145 - 2.137) | (0.192 - 4.236) | (0.402 - 1.767) |
| Richer | 0.171** | 1.037 | 0.217 | 0.841 |
|  | (0.047 - 0.626) | (0.254 - 4.226) | (0.0308 - 1.530) | (0.423 - 1.673) |
| Richest | 0.371 | 0.502 | 1.497 | 0.648 |
|  | (0.119 - 1.150) | (0.118 - 2.136) | (0.408 - 5.492) | (0.306 - 1.373) |
| Government grant | 1.708 | 0.462 | 1.071 | 1.306 |
|  | (0.741 - 3.933) | (0.199 - 1.075) | (0.359 - 3.192) | (0.762 - 2.238) |
| Constant | 0.067* | 0.185 | 0.407 | 0.919 |
|  | (0.004 - 1.023) | (0.00422 - 8.134) | (0.0397 - 4.164) | (0.065 - 12.940) |
|  |  |  |  |  |
| Observations | 634 | 227 | 564 | 654 |

*Note*. *p < 0.05 **p < 0.01 ***p < 0.001. All outcome variables are dichotomous. Weights are used to account for the probability of selecting the enumeration area, the household in the enumeration area, and the individual in the household, adjusted for nonresponse.

**Alternative cut-offs for the depression variable**

**Table 7** Social support, women 18-29 years. Adjusted odds ratios and 95% confidence intervals.

|  |  |  |
| --- | --- | --- |
| VARIABLES | Depression cut-off 10 | Depression cut-off 12 |
|  |  |  |
| **Social support** |  |  |
| Tangible | 0.530* | 0.430** |
|  | (0.306 - 0.918) | (0.241 - 0.767) |
| Educational | 0.499 | 0.213* |
|  | (0.146 - 1.704) | (0.059 - 0.775) |
| Emotional | 0.471 | 0.539 |
|  | (0.191 - 1.164) | (0.199 - 1.465) |
| Educational and Emotional | 0.853 | 0.944 |
|  | (0.335 - 2.172) | (0.340 - 2.616) |
| Tangible and Educational | 0.332* | 0.392 |
|  | (0.128 - 0.858) | (0.144 - 1.063) |
| Tangible and Emotional | 0.648 | 0.684 |
|  | (0.346 - 1.212) | (0.363 - 1.291) |
| All forms of support | 0.697 | 0.748 |
|  | (0.403 - 1.203) | (0.445 - 1.258) |
| Depression cut-off 10 at baseline | 0.804 |  |
|  | (0.519 - 1.246) |  |
| Depression cut-off 12 at baseline |  | 1.071 |
|  |  | (0.611 - 1.876) |
| **Age** |  |  |
| 21-23 years | 1.099 | 1.063 |
|  | (0.796 - 1.517) | (0.715 - 1.580) |
| 24-26 years | 1.002 | 0.847 |
|  | (0.678 - 1.482) | (0.507 - 1.416) |
| 27-29 years | 1.002 | 0.837 |
|  | (0.659 - 1.523) | (0.486 - 1.439) |
| **Education** |  |  |
| Primary | 0.598 | 0.364 |
|  | (0.149 - 2.397) | (0.060 - 2.200) |
| Incomplete secondary | 0.605 | 0.532 |
|  | (0.205 - 1.787) | (0.188 - 1.509) |
| Completed secondary | 0.641 | 0.409 |
|  | (0.235 - 1.750) | (0.156 - 1.071) |
| Tertiary | 0.607 | 0.450 |
|  | (0.189 - 1.943) | (0.131 - 1.544) |
| Single | 1.175 | 0.860 |
|  | (0.641 - 2.151) | (0.450 - 1.645) |
| Away from home | 0.754 | 0.879 |
|  | (0.442 - 1.287) | (0.499 - 1.547) |
| Size of meal cut last 5 days | 0.995 | 1.149 |
|  | (0.639 - 1.551) | (0.634 - 2.081) |
| **Wealth index quintile** |  |  |
| Poorer | 0.696 | 0.551* |
|  | (0.425 - 1.138) | (0.311 - 0.979) |
| Middle | 0.999 | 0.949 |
|  | (0.630 - 1.583) | (0.505 - 1.786) |
| Richer | 0.951 | 0.867 |
|  | (0.601 - 1.504) | (0.473 - 1.590) |
| Richest | 0.920 | 0.854 |
|  | (0.579 - 1.462) | (0.472 - 1.543) |
| Government grant | 1.147 | 1.259 |
|  | (0.789 - 1.666) | (0.808 - 1.963) |
| Constant | 1.197 | 0.993 |
|  | (0.355 - 4.033) | (0.275 - 3.590) |
|  |  |  |
| Observations | 1,432 | 1,432 |

*Note*. *p < 0.05 **p < 0.01 ***p < 0.001. All outcome variables are dichotomous. Weights are used to account for the probability of selecting the enumeration area, the household in the enumeration area, and the individual in the household, adjusted for nonresponse

**A condensed measure of support**

**Table 8** Social support, women 18-29 years. Adjusted odds ratios and 95% confidence intervals. The reference group is no support.

| Outcome | Tangible only or educational and/or emotional support | | | Educational /Emotional support only | |
| --- | --- | --- | --- | --- | --- |
| **Sexual practices** |  |  |  | |  |
| More than one sex partner | 0.35** | [0.16 - 0.74] | 0.69 | | [0.10 - 4.88] |
| Consistent condom use | 0.92 | [0.55 - 1.52] | 0.57 | | [0.17 - 1.88] |
| Sex after alcohol use | 0.39*** | [0.24 - 0.64] | 0.91 | | [0.40 - 2.04] |
| Sex after drug use | 0.30* | [0.10 - 0.88] | 2.14 | | [0.33 - 13.77] |
| **Health behavior** |  |  |  | |  |
| Alcohol use | 0.38*** | [0.24 - 0.60] | 0.99 | | [0.48 - 2.07] |
| Drug use | 0.21** | [0.08 - 0.57] | 0.87 | | [0.15 - 5.18] |
| HIV tested | 1.20 | [0.69 - 2.08] | 1.38 | | [0.54 - 3.52] |
| **Health** |  |  |  | |  |
| Depression | 0.46*** | [0.31 - 0.70] | 0.64 | | [0.27 - 1.53] |
| HIV status | 0.72 | [0.31 - 1.68] | 1.13 | | [0.27 - 4.63] |
| Sexually transmitted infection | 1.01 | [0.38 - 2.65] | 1.32 | | [0.28 - 6.22] |

*Note*. Estimated odds ratios and 95% confidence intervals are in brackets. The number of observations in the models ranges from 953 to 1,700. Logistic regressions were used to estimate the odds ratios. All models are adjusted for prior values of three-year age dummies, educational attainment, household assets, marital status, size of the meal cut last five days, away from home for more than a month during the last 12 months, and number of government grants received, assessed in the baseline survey. Each model also controls for prior values of the respective outcomes assessed in the baseline survey, except HIV status since all women in the follow-up survey were HIV negative. *p < 0.05 **p < 0.01 ***p < 0.001.
